# Stigma and Mental Health of Sexual Minority Women Former Victims of Intimate Partner Violence

**DOI:** 10.1177/08862605211072180

**Published:** 2022-02-22

**Authors:** Emma Fedele, Robert-Paul Juster, Stéphane Guay

**Affiliations:** 1 Master’s candidate in Criminology, School of Criminology, 5622 University of Montreal, Montreal, QC, Canada; 2 Assistant Research Professor, Department of Psychiatry and Addiction, University of Montreal, Montreal, QC, Canada; 3 Tenured Professor, School of Criminology and Department of Psychiatry and Addiction, University of Montreal, Montreal, QC, Canada

**Keywords:** criminology, domestic violence, mental health and violence, intimate partner violence, minority stress, LGBTQ, violence against LGBTQ, sexual minority women

## Abstract

Sexual minority women (SMW) are at high risk of experiencing stigma, mental health problems, and being victims of intimate partner violence (IPV). This vulnerability can be explained by the sexual and gender minority stress model, stating that sexual and gender minority people suffer from specific stress factors added to general stressors, leading to more mental health and relationships problems. OBJECTIVE: The main goal of this study was to assess the impact of minority stress factors and former IPV victimization on the current mental health of Canadian SMW, as a function of their sexual and gender identity. METHOD: In total, 209 individuals identifying as women (M age = 33.9), living in Canada and who lived in a past violent relationship with a woman responded to an online survey. Well-validated questionnaires assessed sexual orientation and gender identity, former IPV behaviors, minority stress factors, depression, and anxiety. RESULTS: Hierarchical regressions showed that past psychological aggression was positively associated with anxiety symptoms and past sexual coercion with depressive symptoms. Not being monosexual was also associated more severe symptoms of depression and age was negatively associated with the severity of anxiety symptoms. After controlling for age, race/ethnicity, sexual and gender identity and former IPV victimization, having negative feelings about being a SMW was strongly associated with both depression and anxiety symptoms. CONCLUSION: These results provide new information on the interconnected associations between former IPV, minority stress and SMW's mental health. Findings highlight the need to adapt clinical interventions to help buffer against victimization faced by IPV victims who identify as sexual and gender minorities.

Lesbian, gay, bisexual, transgender, and queer (LGBTQ+) individuals, particularly sexual minority women (SMW), are at an increased risk of mental health problems (Björkenstam et al., 2017; Gonzales et al., 2016) and intimate partner violence (IPV) victimization (Edwards et al., 2015; Goldberg & Meyer, 2013). IPV victimization can include physical, sexual, emotional abuse, and controlling behaviors by an intimate partner or ex-intimate partner (World Health Organization, 2012). The minority stress model (Meyer, 2003) explains this vulnerability by the fact that sexual and gender minorities suffer from unique, chronic, social, and structural forms of stigma and stress that are in addition to general stress factors. The link between IPV victimization and mental health in the LGBTQ+ population is still understudied compared to research on heterosexual relationships (Gehring & Vaske, 2017). Using the minority stress model, this study contributes to the research on LGBTQ+ victims of IPV by investigating depression and anxiety in SMW victims of past same-gender IPV, and the specific stress factors that influence associations.

## Minority Stress Theory, Mental Health, and IPV

The sexual and gender minority stress model defined by Meyer (2003) identifies four categories of minority stress factors: (1) *external prejudice* such as prejudice events, discrimination or nonevents, defined as “anticipated events or experiences that do not come to pass” (Frost & Meyer, 2017, p. 1196); (2) *stigma consciousness,* described as expecting “negative regards from members of the dominant culture” (Frost & Meyer, 2017, p. 1196); (3) *outness degree* or *identity concealment*; and (4) *internalized homophobia,* defined as the internalization of negative social value about one’s sexuality (Frost & Meyer, 2017; Meyer, 2003).

This model was primarily created to understand the vulnerability of the LGB (lesbian, gay, and bisexual) community to mental health problems: stigmatization gives rise to alienation, a lack of integration to the community, as well as self-acceptance difficulties and is positively associated with depressive symptoms, substance use and suicidal ideation (Meyer, 2003). Since then, this model was enriched and validated with different samples to be more inclusive of gender diversity (Testa et al., 2014). These models show direct and indirect associations between proximal and distal minority stress factors and general anxiety disorder, social anxiety, depression, and substance use (Dyar & London, 2018; Lehavot & Simoni, 2011; Mahon et al., 2021), as well as IPV victimization and perpetration (Badenes-Ribera et al., 2019; Balsam & Szymanski, 2005).

## Mental Health in the LGBTQ+ Community

Since the 2000s, the number of studies of mental health among the LGBTQ+ community or including LGBTQ+ people has increased. Multiple national American surveys using representative samples (Gonzales et al., 2016; López et al., 2021) show that gay men, lesbian women, and bisexual women and men suffer from more psychological distress, depressive symptoms, panic attacks, generalized anxiety disorder, or substance use problems than heterosexual people. Gender diverse individuals also seem to be more vulnerable than cisgender individuals to generalized and social anxiety, depression, psychological distress, eating disorders and suicidal ideation (Lefevor et al., 2019). In particular, bisexual women (Björkenstam et al., 2017; Dyar & London, 2018) and genderqueer people (who do not conform to the gender binary) (Lefevor et al., 2019) seem to be two of the most vulnerable subgroups to mental health problems.

## IPV Victimization and Mental Health

IPV victimization has multiple consequences, including mental illness. The World Health Organization reports that women who are abused suffer higher levels of depression, anxiety, phobias, emotional distress, thoughts of suicide and attempted suicide as well as alcohol and drug abuse, eating and sleep disorders, physical inactivity, poor self-esteem, post-traumatic stress disorder, smoking, self-harm, and unsafe sexual behaviors (World Health Organization, 2012). Physical violence is the most studied kind of IPV, but research highlights the unique contribution of psychological abuse and stalking behaviors to the prediction of depression as well (Lagdon et al., 2014; Mechanic et al., 2008) and the association of sexual IPV with greater risks of PTSD, depressive symptoms, problematic substance use, and suicidality (Barker et al., 2019).

These associations are potentially amplified for members from the LGBTQ+ community (Gehring & Vaske, 2017; Hellemans et al., 2015; McKenry et al., 2006), particularly for women (McKenry et al., 2006). To date, the majority of IPV studies do not include LGBTQ+ people (Gehring & Vaske, 2017), and therefore do not include SMW. This omission exists despite the fact that SMW are one of the most vulnerable subgroups to IPV victimization (Ard & Makadon, 2011).

## IPV in the LGBTQ+ Community

Research on IPV prevalence in the LGBTQ+ community reports inconsistent results. Studies vary greatly according to how IPV is defined and operationalized, the timeframe used, and the sampling methods (Badenes-Ribera et al., 2015; Gehring & Vaske, 2017). Many studies are also prone to not take into account the heterogeneity of the LGBTQ+ community or to confuse sexual identity and behavior, which can bias results (Badenes-Ribera et al., 2015). Sexual identity can be defined as the group or sub-group people identify as in regard to their sexual orientation, whereas sexual behavior is based on the gender of their sexual or romantic partner. Those two concepts are not interchangeable as some people can have homosexual behaviors without identifying themselves as part of the LGBTQ+ community (McKenry et al., 2006) and vice versa. Moreover, orientation is not fixed throughout life, meaning that you cannot assume the gender of someone based on the sexual identity of their partner (Badenes-Ribera et al., 2015).

Despite these problems, nationally representative surveys show that SMW are more vulnerable to IPV than other groups (Ard & Makadon, 2011; Goldberg & Meyer, 2013; Walters et al., 2013). As an example, the National Intimate Partner and Sexual Violence Survey showed that, on 16 507 respondents, 61.1% of bisexual women, 43.8% of lesbian women, and 35% of heterosexual women reported experiencing rape, physical violence, and/or stalking within an intimate relationship at least once in their lifetime (Walters et al., 2013). Moreover, same-gender IPV can take specific forms, like identity abuse, a “set of tactics of IPV that leverage heterosexism and cissexism against LGBTQ survivors” (Woulfe & Goodman, 2021). It can manifest itself as experiences of threat to reveal one’s partner’s sexual orientation, or as a behavior that reinforces internalized homophobia, like telling one’s partner that they need to be punished because of their homosexual behaviors (Ard & Makadon, 2011; Badenes-Ribera et al., 2015; Kimmes et al., 2019). These unique forms of violence are generally not included in studies (Badenes-Ribera et al., 2015) since classic IPV measures like the CTS-2 (Straus et al., 1996) do not take them into account.

## Research Goals

The main goal of this study was to assess the impact of minority stress factors and different forms of past IPV victimization on the mental health of Canadian sexual minority women (SMW), as a function of their sexual and gender identity. Based on our literature review, we hypothesized that physical, psychological, sexual, and identity abuse would have a significant effect on depression and anxiety symptoms. We also hypothesized that minority stress would be positively associated with depression and anxiety symptoms, even when controlling for IPV victimization. We divided SMW into subgroups using spectrums of sexual orientation and gender identity to distinguish between lesbian or gay women, SMW who are not strictly attracted by women (bisexual, pansexual, queer women or else), cisgender women, and gender diverse women. We also measured age and race/ethnic identity as potential covariates.

## Materials and Methods

### Participants

Recruitment promotion material was sent to LGBTQ+ organizations and posted on Facebook groups, listservs and via Instagram, Twitter, and Reddit. Posts were labeled as follows: “Study on the impact of minority stress on intimate partner violence and conflicts in female relationships.” The inclusion criteria for our study were stated in the posters as such: participants must identify as women or transfeminine, have experienced IPV in a past relationship with a woman, be 18 or older, live in Canada, and speak English or French. There was no time frame in which the violent relationship must have ended. We included every person who answered our survey in its entirety, regardless of their sexual or gender identity, as they all had a past relationship involving IPV with a woman. From the 400 individuals who entered our survey, 209 completed it in its entirety.

### General Protocol

This study was approved by the *So*ciété e*t Culture* Research Ethics Committee (CER-SC) of the University of Montreal. Participants were invited to participate in the online survey from 11/2020 to 04/2021. Individuals first provided their consent, by checking that they read our electronic information and consent form and that they agreed to participate in the research project. Participants had to respond to two security questions that served as exclusion criteria. If a respondent answered that they currently lived in a violent relationship or that they were not able to complete the questionnaire in private, without any risk for their well-being, the questionnaire automatically closed itself and they were referred to helping organizations. After completing the questionnaire, participants had the opportunity to give us their email address to be included in a random draw and had a chance to win one of five CAD 50 prepaid Visa credit cards.

### Questionnaires

Questionnaires were split into five sections and available in English or French. If no validated French version was available, we translated the English version of the tool using a back translation method. Questionnaires were completed electronically via Survey Monkey, a secured web-based questionnaire interface. The median value of time of completion was 19 minutes for participants who completed it in its entirety.

#### Sexual and Gender Identity

Gender identity was measured with the 3-item Multidimension sex/gender measure (Bauer et al., 2017), a trans-inclusive measure assessing sex assigned at birth, current gender identity (which included cultural gender minorities) and the gender participants currently live as there day-to-day life. This measure was recommended by the authors for population survey meant for multi-purpose analysis as a one-item question might under-identify trans respondents (Bauer et al., 2017). Sexual identity was measured by asking participants how they identified themselves on a scale ranging from (1) exclusively heterosexual to (7) exclusively homosexual and (8) asexual and (9) other.

#### Current Sexual Minority Stress

All instruments on sexual minority stress assessed it at the time of completion. Prejudice events were measured using an updated version (Arambula, 2015) of the Heterosexist, Harassment, Rejection and Discrimination Scale (Szymanski, 2006). This 14-item scale measures rejection and harassment, discrimination at school and work, and other discriminations on a scale ranging from (0) It never happened to (6) More than 20 times. This instrument showed good internal consistency in our sample (α = 0.90).

Stigma consciousness was measured using an adapted version of the Stigma Consciousness Questionnaire for Gay Men and Lesbians (Pinel, 1999). This 10-item questionnaire measures how much an individual thinks they are judged on the basis of a stereotype on a 6-point Likert scale and showed good internal consistency in our sample (α = 0.85).

Concealment was evaluated using an outness measure (Meyer et al., 2002). Participants were asked to indicate who they were “out” to about their sexual orientation using a 4-point Likert scale. This instrument showed good internal consistency in our sample (α = 0.83).

Internalized homophobia was measured using the 13-item Connection to the Community, 16-item Public Identification as Lesbian, 8-item Personal Feelings About Being a Lesbian, and 8-item Attitudes Toward Other Lesbian subscales of the Lesbian Internalized Homophobia Scale (LIHS) (Szymanski & Chung, 2001). Questions were adapted to include all SMW. Each subscale showed acceptable internal consistency in our sample (αs varied between 0.79 and 0.89). The Public Identification as a SMW subscale was removed from our analysis because of collinearity with the Concealment variable (variance inflation factor for Concealment was 2.9 when the subscale was integrated to our model and 1.4 when it was dropped).

#### Past IPV Victimization

First, basic information on the former violent relationship were asked concerning the length of the relationship and the time since it has ended. Then, past IPV victimization was measured using the Revised Conflict Tactics Scales—Short Form (CTS-2S; M. A. Straus & Douglas, 2004), measuring negotiation, physical assault, injury, psychological aggression and sexual coercion perpetration, and victimization and their severity. Each 20 items are measured on a scale ranging from (0) It has never happened to (6) More than 20 times. The CTS-2S (Straus & Douglas, 2004) is less hetero-cis-centric than the CTS-2 (Straus et al., 1996) since it does not differentiate between types of sexual interaction. However, the term “condom” was replaced by the term “protection” to make it appropriate for SMW. Identity abuse was measured by creating two other items, asking if the participant or their partner had threatened to tell their friends, family, and colleges (or other institution) about their sexuality.

#### Current Depression and Anxiety

Depression and anxiety symptoms were respectively measured using the Patient Health Questionnaire—9 (Kroenke et al., 2001) and the Generalized Anxiety Disorder—7 (Spitzer et al., 2006). These instruments measure depression and anxiety symptoms over the last 2 weeks on a 4-point Likert scale ranging from (0) Never to (3) Nearly every day. Both instruments showed good internal consistency in our sample (αs = 0.91).

### Statistical analysis

Age (in years) and race/ethnic identity were used as covariates. Race/ethnic identity was categorized as follows in our descriptive statistics: (1) White (2) Black (3) Indigenous/First Nations (4) Asian (5) Arab/Maghreb (6) Hispanic (7) other (8) mixed race/ethnicity. It was then dichotomized as (0) White and (1) person of color to be incorporated in our analyses. Variables measuring the frequencies of the different forms of IPV victimization were computed by summing items for each subscale, creating three continuous variables ranging from 0 to 12 for physical, sexual, and psychological victimization. Identity abuse was dichotomized as it was too skewed to be incorporated in our analysis as a continuous variable. For mental health and minority stress, variables were computed by summing items together to create a final score for each screening tool, except for the LIHS, where we created three different variables since the subscales are “distinct but correlated dimensions” (Szymanski & Chung, 2001, p. 48) and did not show good enough internal consistency when summed together (α = 0.68).

Preliminary analyses consisted of Pearson’s and Point-Biserial correlations to explore associations among study variables. Our main analyses employed two hierarchical regressions, with depression and anxiety symptoms as dependent variables. In each regression, age, and being a person of color were entered first (Model 1). Being gender diverse and not being strictly attracted to women were entered next (Model 2). Third, frequencies of physical, psychological and sexual victimization, and the dichotomized measure of identity abuse were entered to account for intimate partner victimization (Model 3). Fourth, prejudice events, stigma consciousness, degree of concealment, degree of community connectedness, negative feelings about being a SMW, and negative attitudes towards other SMW were entered to account for minority stress (Model 4). All statistical analyses used SPSS Version 26.

## Results

### Preliminary Analysis

Among our 209 participants 80.9% were White, 2.4% were Black, 1.4% were Indigenous/First Nations, 2.4% were Asian, 1.4% were Middle Eastern, 1.4% were Hispanic, and 10% were mixed race/ethnicity. The mean age of our sample was 33.9, ranging from 19 to 68. Regarding sexual and gender identity, 36.8% were not strictly attracted to women (they identified as bisexual, heterosexual or other) and 27.8% were gender diverse. Seventy-six percent of the participants had been out of their violent relationship for more than 1 year and these relationships lasted on average 38.3 months. 70% had been victims of physical assault, 48.3% of sexual coercion, 98.6% of psychological aggression, and 20.1% of identity abuse. Table 1 lists the sample’s descriptive statistics for demographics. Table 2 provides study variable statistics and their correlation coefficients for descriptive purposes.

**Table 1.** Sample Descriptive Statistics.

Variables	Information
*N*	209
Age, *M* (SE)	33.9 (9.8)
Race/Ethnicity, %	
White	80.9
Black	2.4
Indigenous	1.4
Asian	2.4
Arab/Maghreb	1.4
Hispanic	1.4
Multi-racial	10.0
Region, %	
Alberta	13.4
British Columbia	9.6
Manitoba	0.5
New Brunswick	1.0
Newfoundland and Labrador	1.0
Nova Scotia	2.4
Ontario	22.5
Quebec	42.1
Saskatchewan	7.2
Relationship length (in months), *M* (SE)	38.3 (34.9)
Separation, %	
Less than a month	4.3
2–6 months	8.1
7–12 months	11.5
More than a year	76.1

**Table 2.** Descriptive Statistics and Correlation Coefficients of All Study Variables.

Variable	*M* (SD)	Correlations															
		1	2	3	4	5	6	7	8	9	10	11	12	13	14	15	16
1.Age	33.9 (9.8)	_															
2.Person of color (0–1)	.19 (.39)	−.133															
3.Gender diverse (0–1)	.28 (.45)	−.108															
4.Not strictly lesbian (0–1)	.37 (.48)	−.168*															
5.Homophobic victimization (0–1)	.20 (.40)	−.023	_	_	_	_	_										
6.Physical victimization	3.83 (3.83)	.045	.105	.070	.042	.057	_										
7.Psychological victimization	7.69 (3.19)	.058	.090	.057	.062	.090	.609** (.628**)	_									
8.Sexual victimization	2.30 (3.17)	−.200** (−.217**)	.047	.192**	.201**	.255**	.350** (.250**)	279** (.260**)	_								
9.Prejudice events	2.08 (1.04)	.018	−.044	.373**	−.024	.243**	.079	.070	.103	_							
10.Stigma consciousness	4.60 (1.21)	−.226**	.036	.221**	.204**	.138*	−.022	−.024	.144*	.445**	_						
11.Concealment	1.64 (.67)	−.103	.007	−.049	.318**	.151*	.035	.099	.247** (.180**)	−.187*	.099	_					
12.Community link	2.47 (.61)	−.057	.071	−.078	−.077	.021	.058	.096	−.075	−.081	−.102	.182** (.166*)	_				
13. Negative feelings	1.60 (.69)	−.050	−.031	.058	.149*	.131	.062	−.009 (.043)	.099	.099	.176* (.148*)	.339** (.330**)	.368** (.408**)	_			
14.Negative attitudes	1.56 (.59)	.124 (.151*)	.181**	−.119	−.121	.021	.130	.005	−.078	.021	−.162*	.047	.294** (.292**)	.233** (.246**)	_		
15.Depressive symptoms	9.89 (6.96)	−.204**	.039	.122	.244**	.146*	.061	.107	.265** (.265**)	.099	.186*	.173* (.152*)	.034	.299** (.279**)	.031	_	
16.Anxiety symptoms	8.67 (5.81)	−.239**	.032	.098	.205*	.150*	.077	.184**	.248** (.256**)	.146*	.233**	.076 (.047)	−.012	.242** (.177*)	−.021 (−.022)	.710**	_

*p* < .05. **p < .01.

Scores between two continuous variables are Pearson’s correlations. Scores in brackets are Spearman’s correlation and appear when one of Pearson’s correlations’ assumption was violated. Scores between a continuous variable and a dichotomous variable (0–1) are Point Biserial correlation.

### Main analysis

Two hierarchical regressions were employed in four steps: Model 1 contained only covariates; Model 2 contained covariates and subgroups; Model 3 contained covariates, subgroups, and IPV victimization; and Model 4 contained covariates, subgroups, victimization, and minority stress factors. All tests of multicollinearity were in an acceptable range, as determined by variance inflation factors ranging from 1.02 to 1.78 for depression and anxiety symptoms.

The first regression investigated the degree to which covariates, subgroups, former IPV victimization and minority stress factors were associated with depressive symptoms (Table 3). Collectively, the variables accounted for 20% of the variance in depressive symptomology (F_(14,194)_ = 3.53, *p* < .01, *R*^*2*^ = .20). Covariates (age and being Non-White) accounted for 4% of the variance (F_(2;206)_ = 4.51, *p* < .05, *R*^*2*^ = .04). Subgroups (being non-monosexual and being gender diverse) accounted for an additional 5% of variance (F_(4;204)_ = 4.20, *p* < .01, *R*^*2*^ = .09, Δ*p* =.005, Δ*R*^*2*^ = .05). Past IPV victimization explained 14% of the variance (F_(8;200)_ = 3.92, *p* < .01, *R*^*2*^ = .14), accounting for an additional 5% of variance (Δ*p* = .038, Δ*R*^*2*^ = .05). Minority stress accounted for an additional 7% of the variance (Δ*p* =.014, Δ*R*^*2*^ = .07). In the final model, being non-monosexual (β = .15, *p* < .05), having been sexually victimized (β = .17, *p* < .05) and having negative feelings about being a SMW (β = .27, *p* < .01) were positively associated with depressive symptoms.

**Table 3.** Regression Coefficients Predicting Depressive Symptoms.

Model	*R*^2^	F	df1; df2	Sig	Predictors	B	t	Sig
1	.042	4.507	2; 206	.012	Age	−.203	−2.947	.004
					Person of color	.012	.179	.858
2	.091	5.090	4; 204	.001	Age	−.160	−2.325	.021
					Person of color	.031	.456	.649
					Gender diverse sub-group	.056	.809	.420
					Not strictly attracted to women	.206	2.947	.004
3	.136	3.921	8; 200	.000	Age	−.138	−1.979	.049
					Person of color	.005	.071	.943
					Gender diverse sub-group	.020	.284	.777
					Not strictly attracted to women	.171	2.462	.015
					Homophobic victimization	.073	1.034	.302
					Physical victimization	−.058	−.671	.503
					Psychological victimization	.082	.981	.328
					Sexual victimization	.178	2.336	.020
4	.203	3.532	14; 194	.000	Age	−.131	−1.872	.063
					Person of color	.024	.340	.734
					Gender diverse sub-group	−.004	−.054	.957
					Not strictly attracted to women	.152	2.086	.038
					Homophobic victimization	.033	.465	.643
					Physical victimization	−.085	−.995	.321
					Psychological victimization	.109	1.315	.190
					Sexual victimization	.168	2.187	.030
					Prejudice events	.029	.348	.728
					Stigma consciousness	.038	.479	.633
					Concealment	−.024	−.312	.756
					Link to the community	−.052	−.709	.479
					Negative feelings about being a SMW	.265	3.369	.001
					Negative attitudes towards other SMW	.020	.271	.787

The second regression investigated the degree to which covariates, subgroups, former IPV victimization, and minority stress factors were associated with anxiety symptoms (Table 4). Collectively, the variables accounted for 22% of the variance in anxiety symptomology (F_(14,194)_ = 3.98, *p* < .01, *R*^*2*^ = .22). Covariates (age and being Non-White) accounted for 6% of the variance (F_(2,206)_ = 6.22, *p* > .01, *R*^*2*^ = .06). Subgroups (being non-monosexual and being gender diverse) accounted for an additional 3% of variance (F_(4,204)_ = 4.83, *p* > .01, *R*^*2*^ = .09, Δ*p* =.04, Δ*R*^*2*^ = .03). Past IPV victimization explained 15% of the variance (F_(8,200)_ = 4.41, *p* < .01, *R*^*2*^ = .15), accounting for an additional 6% of variance (Δ*p* =.006, Δ*R*^*2*^ = .06). And finally, minority stress factors accounted for an additional 7% of variance (Δ*p* =.007, Δ*R*^*2*^ = .07). In the final model, age was negatively associated with anxiety symptoms (β = −.19, *p* < .01), whereas psychological victimization (β = .24, *p* < .01) and having negative feelings about being a SMW (β = .25, *p* < .01) were positively associated with more anxiety symptoms.

**Table 4.** Regression Coefficients Predicting Anxiety Symptoms.

Model	*R*^2^	F	df1; df2	Sig	Predictors	β	t	Sig
1	.057	6.223	2; 206	.002	Age	−.239	−3.496	.001
					Person of color	.001	.009	.993
2	.086	4.829	4; 204	.001	Age	−.205	−2.983	.003
					Person of color	.015	.226	.822
					Gender diverse sub-group	.038	.544	.587
					Not strictly attracted to women	.163	2.324	.021
3	.150	4.411	8; 200	.000	Age	−.198	−2.878	.004
					Person of color	−.022	−.326	.745
					Gender diverse sub-group	.001	.016	.987
					Not strictly attracted to women	.126	1.821	.070
					Homophobic victimization	.093	1.315	.190
					Physical victimization	−.092	−1.078	.283
					Psychological victimization	.200	2.408	.017
					Sexual victimization	.137	1.807	.072
4	.223	3.978	14; 194	.000	Age	−.186	−2.701	.008
					Person of color	.003	.045	.964
					Gender diverse sub-group	−.059	−0.813	.417
					Not strictly attracted to women	.135	1.885	.061
					Homophobic victimization	.058	.818	.414
					Physical victimization	−.119	−1.414	.159
					Psychological victimization	.236	2.882	.004
					Sexual victimization	.136	1.800	.073
					Prejudice events	.044	.527	.599
					Stigma consciousness	.091	1.152	.251
					Concealment	−.122	−1.613	.108
					Link to the community	−.086	−1.185	.237
					Negative feelings about being a SMW	.254	3.268	.001
					Negative attitude with other SMW	−.003	−.035	.972

To summarize, Model 4 integrating covariates, subgroups, victimization variables, and minority stress factors was the best fit for depressive symptoms as well as anxiety symptoms for which it, respectively, explained 20% and 22% of the variance. For both dependent variables, adding minority stress factors added a significant part of variance. In these models, intensity of negative feelings about being a SMW was associated with depression and anxiety symptoms even after controlling for past IPV victimization, which itself was associated with depression and anxiety symptoms. Age was also negatively associated with anxiety symptoms, and identifying as bisexual, pansexual, queer, or else was positively associated with depressive symptoms.

## Discussion

**Figure 1.**
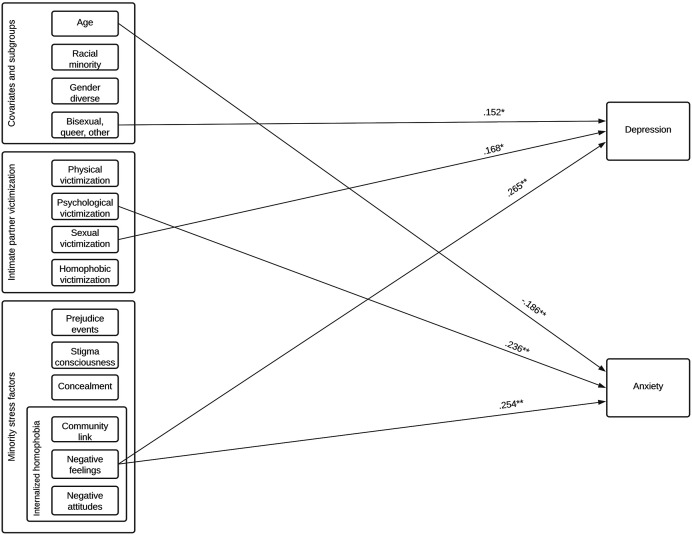
Theoretical model and significant associations between covariates, subgroups, past victimization, minority stress, and depressive and anxiety symptoms.

This study assessed the impact of minority stress factors and past IPV victimization on symptoms of depression and anxiety among Canadian SMW who experienced past IPV. Significant results are shown in Figure 1. Our results partly support our first hypothesis that physical, psychological, sexual, and identity abuse would have a subsequent impact on depression and anxiety. Indeed, we found that past psychological and sexual victimization were significantly associated with current levels of symptoms of anxiety and depression respectively, which is in accordance with previous literature on different-gender relationships (Barker et al., 2019; Lagdon et al., 2014; Mechanic et al., 2008). This suggests that the association between IPV and mental health problems also exists in the LGBTQ+ community (Gehring & Vaske, 2017; Hellemans et al., 2015; McKenry et al., 2006).

The absence of significant results for physical victimization is in accordance with Hellemans et al. (2015), who found a significant association between psychological IPV and mental health in LGBTQ+ people, but not with physical violence and mental health. A potential explanation is that psychological IPV is more mentally damaging than physical IPV because it attacks the victim’s sense of self-esteem and ability to feel good about themselves, whereas physical violence comes from a problem inside the perpetrator, a possibility which has already been discussed in heterosexual and cisgender relationships (Follingstad et al., 1990; Hellemans et al., 2015). However, individuals in our sample all suffered from at least one form of past IPV, and nearly all our sample suffered from psychological violence, which might have altered our results. Having a control group of individuals who did not suffer from any kind of IPV in the past might be better fitted to compare and analyze the associations between different types of IPV and mental health. Furthermore, we dichotomized our variable of identity abuse because our distribution was skewed, which might have further distorted the results (Irwin & McClelland, 2003). In the future, a larger sample with a control group and a more refined assessment of identity abuse might counter this effect and could allow us to split individuals into different groups depending on the kind of victimization they suffered from with consideration about severity and time courses.

Our findings also support our second hypothesis that minority stress is positively associated with depression and anxiety symptoms, even when controlling for IPV victimization. This is in accordance with previous research (Dyar & London, 2018; Lehavot & Simoni, 2011; Mahon et al., 2021; Meyer, 2003) and highlights the importance of same-gender specific stressors on mental health, once again confirming the minority stress theory. It is noteworthy that internalized homophobia (IH) was the only minority stress factor significantly associated with depression and anxiety symptoms, suggesting that proximal stressors have more impact on mental health than prejudice events. This importance of proximal stressors was previously highlighted when studying minority stress and IPV (Badenes-Ribera et al., 2019; Edwards & Sylaska, 2013). A meta-analysis on IPV risk markers in same-gender relationships found that internalized homophobia was the strongest same-gender-specific risk marker for IPV victimization in women (Kimmes et al., 2019). However, they also found that research on same-gender-specific risk markers of IPV was extremely limited (Kimmes et al., 2019). This work therefore contributes to the research on minority stress by highlighting the importance of proximal minority stressors on mental health.

**Figure 2.**
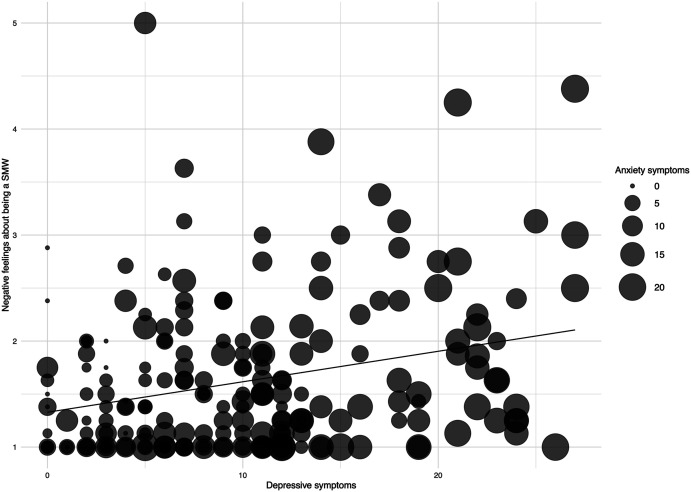
Association between depressive and anxiety symptoms and negative feelings about being a sexual minority women (smw).

In addition, this work distinguishes between different dimensions of IH and shows that having negative feelings about being a SMW have more impact than negative attitudes towards others and lack of community connectedness. This further suggests that internalized negative thoughts are the most detrimental factor to mental health in our study (Figure 2). IH was also the most important factor in both of our regressions. This work provides novel findings suggesting that, in SMW IPV survivors, IH is more important than any other type of victimization when predicting symptoms of depression and anxiety. Nevertheless, other minority stress factors should not be entirely overlooked since preliminary correlation analyses (Table 2) showed significant and positive associations between prejudice events and anxiety symptoms; stigma consciousness and both anxiety and depressive symptoms; and identity concealment and depressive symptoms.

Secondary analyses of covariates revealed that older participants experienced less anxiety symptoms. This is in accordance with previous literature that tends to show that, in Western countries, older adults report fewer mental illness problems and experience more positive mental health (Schönfeld et al., 2017). Being non-monosexual was also associated with more depressive symptoms, once again confirming that the LGBTQ+ community is not a homogenous group (Björkenstam et al., 2017; Dyar & London, 2018). This difference can potentially be explained by the fact that non-monosexual people suffer from stigmatization from both heterosexual and homosexual people (Dyar & London, 2018; Woulfe & Goodman, 2021).

### Limitations

First, like most studies of the LGBTQ+ community (Carvalho et al., 2011), we used a convenience sample. Most of our recruitment was done through LGBTQ+ organizations and social media. This might have impacted our results since people who suffer the most from minority stress, and particularly IH, experience feelings of shame, guilt, and distress regarding their sexuality or gender, making them less likely to be present on LGBTQ+ platforms (Carvalho et al., 2011), where our recruitment took place. Moreover, our study was promoted as an IPV study for transparency, which means that only people who identified as victims of past IPV answered. This cause a self-selection bias and limits our sample, particularly in the LGBTQ+ community where same-gender IPV is not easily openly discussed (Edwards et al., 2015; Gehring & Vaske, 2017).

Second, we used an online survey, which is a good way to reach the LGBTQ+ community (Kubicek, 2018), but has its limits concerning the quantity and quality of measurements. We had to limit our questions so that it was not too time-consuming. Therefore, some concepts known for their associations to both IPV and minority stress, such as PTSD (Barker et al., 2019; World Health Organization, 2012), substance use (Kubicek, 2018) or social support (Meyer, 2003; Testa et al., 2014), and potential mediators and moderators such as relationship satisfaction (Balsam & Szymanski, 2005; Lewis et al., 2014), rumination (Lewis et al., 2014), and resilience (Meyer, 2003, 2015; Testa et al., 2014) were not included in our model. This might explain that it only accounted for 20–22% of our variance. Furthermore, it is based on self-reported measures, meaning that answers are dependent on the participants’ subjective understanding of the questions and their recollection of their behaviors and that of their ex-partners. This can cause informant bias, particularly since IPV events occurred more than a year ago for most of our sample.

Third, we used a cross-sectional design which does not allow us to make assumptions about causality. However, the temporal structure of our data can only allow us to assume that past events predicted current (i.e., past 14 days) mental health. Indeed, mental health and minority stress were measured at the time of the questionnaire completion, but victimization was a past event. We might therefore assume that past IPV impact current mental health, and not vice versa, but a longitudinal study would allow us to validate this assumption and give more information on the evolution of proximal and distal minority stress factors over time.

### Future Directions

Although we did not find any significant association between being gender diverse and mental health, previous research states otherwise (Lefevor et al., 2019; Testa et al., 2014). This absence of significant associations might be due to the fact that our questionnaire was primarily focused on sexual minority stress factors. We included gender specific terms, but we did not include gender-specific stressors. However, literature shows that the experience of gender diverse people can differ from LGB people. They might experience additional forms of discrimination and distal stressors, like non-affirmation, which “occurs when one’s internal sense of gender identity is not affirmed by others” (Testa et al., 2014, p. 66). Moreover, it is harder for gender diverse people to conceal their identity since gender identity is partly guided by physical cues and many languages and cultures use gender as a primary identifying characteristic (Testa et al., 2014). Therefore, future research on sexual and gender minority stress should concentrate more on gender minorities, using a more complete version of the sexual and gender minority stress model (Testa et al., 2014) that might best represent their unique experiences.

Moreover, we adapted a short version (Straus & Douglas, 2004) of the most widely used IPV measure (CTS-2, Straus et al., 1996), to allow for comparison between samples. However, some of the items of the CTS-2 (Straus et al., 1996) are based on a hetero-cis-centric definition of sex as penile-vaginal intercourse which is not culturally appropriate for use in the LGBTQ+ population and does not capture LGBTQ+-specific types of IPV (Dyar et al., 2021). It is problematic as it might miss instances of IPV (Dyar et al., 2021). Some specific measures were created in the past years (Chan & Cavacuiti, 2008; Dyar et al., 2021; Stephenson & Finneran, 2013; Woulfe & Goodman, 2021) and should be used more systematically. Future works should also focus on validating them on different LGBTQ+ populations as development of validated measures is still at its early stage (Badenes-Ribera et al., 2015; Kimmes et al., 2019) and as specific control strategies might vary depending on the ethnic and cultural background of participants.

Finally, despite our efforts to be visible to racial and ethnic minorities, only 40 participants identified as Non-White. Data from the 2015 Canadian Community Health Survey (*N* = 51 545) show that 17.6% of lesbian and 15.1% of bisexual women living in Canada identify as Non-White (Gilmour, 2019). Therefore, our sample is similar in proportion of racial/ethnic minorities of Canadian SMW, albeit small. We regrouped all racial/ethnic minority groups into one covariate, but by doing so, we erased the unique experience of the different racial/ethnic sub-groups regarding minority stress. Moreover, we focused our questionnaire on specific variables that targeted sexual identity alone, but the discrimination someone suffers from may be different based on their intersecting identities (Lewis & Neville, 2015; Nadal et al., 2015). This might explain why we did not find any significant differences between race/ethnic subgroups on mental health and minority stress, despite previous reports to the contrary (López et al., 2021). Future research should use larger and more diverse samples to take into account intersectionality.

### Implications for Policy and Practice

Our results show that many SMW suffer from the same forms of IPV than heterosexual victims: 99% of our sample reported psychological victimization, 70% reported physical victimization and 48% reported sexual victimization. Moreover, 20% reported having been victim of identity abuse, and negative feelings about being a SMW was moderately associated with more depression and anxiety symptoms. This highlights the impact of LGBTQ+ specific variables on SMW IPV survivors and underlines the need to train clinicians to recognize IPV in same-gender relationships, to refer them to the right organizations and to give psychoeducation (Ard & Makadon, 2011; Edwards et al., 2015; Kubicek, 2018).

Intervention should also focus more on mental health, substance use problems, social support and discrimination as those areas are where LGBTQ+ communities are more vulnerable than others (Kubicek, 2018). Prevention is also necessary, by raising awareness to the LGBTQ+ community inclusion difficulties in the general population and by making IPV prevention programs more inclusive and accessible to the LGBTQ+ community (Kubicek, 2018).

## Conclusion

This study provides new information on the associations between past IPV, minority stress and SMW’s mental health. Having negative feelings about being a SMW was associated to more depression and anxiety symptoms; sexual victimization to more depressive symptoms; and psychological victimization and being younger were associated with more anxiety symptoms. Not being strictly attracted to women was also associated with more depressive symptoms. By identifying specific factors of IPV and mental health problems in SMW, this novel work points towards future directions that can assess the needs of SMW, set up appropriate help, and adapt intervention for the specific experience of understudied LGBTQ+ subgroups.

## References

[bibr1-08862605211072180] Arambula C. (2015). Heterosexism, harassment, discrimination, and coping mechanisms among lesbian, gay, bisexual, transgender, and questioning individuals [Ed.D., Texas A&M University Kingsville]. https://www.proquest.com/docview/1780991685/abstract/1E85671685F14419PQ/1

[bibr2-08862605211072180] Ard K. L. Makadon H. J. (2011). Addressing intimate partner violence in lesbian, gay, bisexual, and transgender patients. Journal of General Internal Medicine, 26(8), 930–933. 10.1007/s11606-011-1697-6 21448753 PMC3138983

[bibr3-08862605211072180] Badenes-Ribera L. Frias-Navarro D. Bonilla-Campos A. Pons-Salvador G. Monterde-i-Bort H. (2015). Intimate partner violence in self-identified lesbians: A meta-analysis of its prevalence. Sexuality Research and Social Policy, 12(1), 47–59. 10.1007/s13178-014-0164-7

[bibr4-08862605211072180] Badenes-Ribera L. Sánchez-Meca J. Longobardi C. (2019). The relationship between internalized homophobia and intimate partner violence in same-sex relationships: A meta-analysis. Trauma, Violence, & Abuse, 20(3), 331–343. 10.1177/1524838017708781 29333955

[bibr5-08862605211072180] Balsam K. F. Szymanski D. M. (2005). Relationship quality and domestic violence in women’s same-sex relationships: The role of minority stress. Psychology of Women Quarterly, 29(3), 258–269. 10.1111/j.1471-6402.2005.00220.x

[bibr6-08862605211072180] Barker L. C. Stewart D. E. Vigod S. N. (2019). Intimate partner sexual violence: An often overlooked problem. Journal of Women’s Health, 28(3), 363–374. 10.1089/jwh.2017.6811 30335574

[bibr7-08862605211072180] Bauer G. R. Braimoh J. Scheim A. I. Dharma C. (2017). Transgender-inclusive measures of sex/gender for population surveys: Mixed-methods evaluation and recommendations. PLOS ONE, 12(5), e0178043. 10.1371/journal.pone.0178043 28542498 PMC5444783

[bibr8-08862605211072180] Björkenstam C. Björkenstam E. Andersson G. Cochran S. Kosidou K. (2017). Anxiety and depression among sexual minority women and men in Sweden: Is the risk equally spread within the sexual minority population? The Journal of Sexual Medicine, 14(3), 396–403. 10.1016/j.jsxm.2017.01.012 28202321 PMC6909248

[bibr9-08862605211072180] Carvalho A. F. Lewis R. J. Derlega V. J. Winstead B. A. Viggiano C. (2011). Internalized sexual minority stressors and same-sex intimate partner violence. Journal of Family Violence, 26(7), 501–509. 10.1007/s10896-011-9384-2

[bibr10-08862605211072180] Chan E. Cavacuiti C. (2008). Gay Abuse Screening Protocol (GASP): Screening for abuse in gay male relationships. Journal of Homosexuality, 54(4), 423–438. 10.1080/00918360801991455 18826169

[bibr11-08862605211072180] Dyar C. London B. (2018). Longitudinal examination of a bisexual-specific minority stress process among bisexual cisgender women. Psychology of Women Quarterly, 42(3), 342–360. 10.1177/0361684318768233

[bibr12-08862605211072180] Dyar C. Messinger A. M. Newcomb M. E. Byck G. R. Dunlap P. Whitton S. W. (2021). Development and initial validation of three culturally sensitive measures of intimate partner violence for sexual and gender minority populations. Journal of Interpersonal Violence, 36(15–16), 8824–8851. 10.1177/0886260519846856 PMC6829031 31057032

[bibr13-08862605211072180] Edwards K. M. Sylaska K. M. (2013). The perpetration of intimate partner violence among LGBTQ college youth: The role of minority stress. Journal of Youth and Adolescence, 42(11), 1721–1731. 10.1007/s10964-012-9880-6 23233160

[bibr14-08862605211072180] Edwards K. M. Sylaska K. M. Neal A. M. (2015). Intimate partner violence among sexual minority populations: A critical review of the literature and agenda for future research. Psychology of Violence, 5(2), 112–121. 10.1037/a0038656

[bibr15-08862605211072180] Follingstad D. R. Rutledge L. L. Berg B. J. Hause E. S. Polek D. S. (1990). The role of emotional abuse in physically abusive relationships. Journal of Family Violence, 5(2), 107–120. 10.1007/BF00978514

[bibr16-08862605211072180] Frost D. M. Meyer I. H. (2017). Minority stress. In: The SAGE Encyclopedia of Psychology and Gender (pp. 1195–1198). SAGE Publications.

[bibr17-08862605211072180] Gehring K. S. Vaske J. C. (2017). Out in the open: The consequences of intimate partner violence for victims in same-sex and opposite-sex relationships. Journal of Interpersonal Violence, 32(23), 3669–3692. 10.1177/0886260515600877 26319709

[bibr18-08862605211072180] Gilmour H. (2019). Sexual orientation and complete mental health. Statistics Canada, 30(11), 3–10. 10.25318/82-003-X201901100001-ENG 31747043

[bibr19-08862605211072180] Goldberg N. G. Meyer I. H. (2013). Sexual orientation disparities in history of intimate partner violence: Results from the California Health Interview Survey. Journal of Interpersonal Violence, 28(5), 1109–1118. 10.1177/0886260512459384 23008053

[bibr20-08862605211072180] Gonzales G. Przedworski J. Henning-Smith C. (2016). Comparison of health and health risk Ffctors between lesbian, gay, and bisexual adults and heterosexual adults in the United States: Results From the National Health Interview Survey. JAMA Internal Medicine, 176(9), 1344. 10.1001/jamainternmed.2016.3432 27367843

[bibr21-08862605211072180] Hellemans S. Loeys T. Buysse A. Dewaele A. De Smet O. (2015). Intimate partner violence victimization among non-heterosexuals: Prevalence and associations with mental and sexual well-being. Journal of Family Violence, 30(2), 171–188. 10.1007/s10896-015-9669-y

[bibr22-08862605211072180] Irwin J. R. McClelland G. H. (2003). Negative consequences of dichotomizing continuous predictor variables. Journal of Marketing Research, 40(3), 366–371. 10.1509/jmkr.40.3.366.19237

[bibr23-08862605211072180] Kimmes J. G. Mallory A. B. Spencer C. Beck A. R. Cafferky B. Stith S. M. (2019). A meta-analysis of risk markers for intimate partner violence in same-sex relationships. Trauma, Violence, & Abuse, 20(3), 374–384. 10.1177/1524838017708784 29333967

[bibr24-08862605211072180] Kroenke K. Spitzer R. L. Williams J. B. W. (2001). The PHQ-9: Validity of a brief depression severity measure. Journal of General Internal Medicine, 16(9), 606–613. 10.1046/j.1525-1497.2001.016009606.x 11556941 PMC1495268

[bibr25-08862605211072180] Kubicek K. (2018). Setting an agenda to address intimate partner violence among young men who have sex with men: A conceptual model and review. Trauma, Violence, & Abuse, 19(4), 473–487. 10.1177/1524838016673599 27756778

[bibr26-08862605211072180] Lagdon S. Armour C., & Stringer M. (2014). Adult experience of mental health outcomes as a result of intimate partner violence victimisation: A systematic review. European Journal of Psychotraumatology, 5, 10.3402/ejpt.v5.24794. 10.3402/ejpt.v5.24794. PMC4163751 25279103

[bibr27-08862605211072180] Lefevor G. T. Boyd-Rogers C. C. Sprague B. M. Janis R. A. (2019). Health disparities between genderqueer, transgender, and cisgender individuals: An extension of minority stress theory. Journal of Counseling Psychology, 66(4), 385–395. 10.1037/cou0000339 30896208

[bibr28-08862605211072180] Lehavot K. Simoni J. M. (2011). The impact of minority stress on mental health and substance use among sexual minority women. Journal of Consulting and Clinical Psychology, 79(2), 159–170. 10.1037/a0022839 21341888 PMC4059829

[bibr29-08862605211072180] Lewis J. A. Neville H. A. (2015). Construction and initial validation of the Gendered Racial Microaggressions Scale for Black women. Journal of Counseling Psychology, 62(2), 289–302. 10.1037/cou0000062 25867696

[bibr30-08862605211072180] Lewis R. J. Milletich R. J. Derlega V. J. Padilla M. A. (2014). Sexual minority stressors and psychological aggression in lesbian women’s intimate relationships: The mediating roles of rumination and relationship satisfaction. Psychology of Women Quarterly, 38(4), 535–550. 10.1177/0361684313517866

[bibr31-08862605211072180] López J. D. Duncan A. Shacham E. McKay V. (2021). Disparities in health behaviors and outcomes at the intersection of race and sexual identity among women: Results from the 2011–2016 National Health and Nutrition Examination Survey. Preventive Medicine, 142, 106379. 10.1016/j.ypmed.2020.106379 33347873

[bibr32-08862605211072180] Mahon C. P. Pachankis J. E. Kiernan G. Gallagher P. (2021). Risk and protective factors for social anxiety among sexual minority individuals. Archives of Sexual Behavior, 50(3), 1015–1032. 10.1007/s10508-020-01845-1 33398699

[bibr33-08862605211072180] McKenry P. C. Serovich J. M. Mason T. L. Mosack K. (2006). Perpetration of gay and lesbian partner violence: A disempowerment perspective. Journal of Family Violence, 21(4), 233–243. 10.1007/s10896-006-9020-8

[bibr34-08862605211072180] Mechanic M. B. Weaver T. L. Resick P. A. (2008). Mental health consequences of intimate partner abuse. Violence Against Women, 14(6), 634–654. 10.1177/1077801208319283 18535306 PMC2967430

[bibr35-08862605211072180] Meyer I. H. (2003). Prejudice, social stress, and mental health in lesbian, gay, and bisexual populations: Conceptual issues and research evidence. Psychological Bulletin, 129(5), 674–697. 10.1037/0033-2909.129.5.674 12956539 PMC2072932

[bibr36-08862605211072180] Meyer I. H. (2015). Resilience in the study of minority stress and health of sexual and gender minorities. Psychology of Sexual Orientation and Gender Diversity, 2(3), 209. 10.1037/sgd0000132

[bibr37-08862605211072180] Meyer I. H. Rossano L. Ellis J. Bradford J. (2002). A brief telephone interview to identify lesbian and bisexual women in random digit dialing sampling. Journal of Sex Research, 39, 139–144. 10.1080/00224490209552133 12476246

[bibr38-08862605211072180] Nadal K. L. Davidoff K. C. Davis L. S. Wong Y. Marshall D. McKenzie V. (2015). A qualitative approach to intersectional microaggressions: Understanding influences of race, ethnicity, gender, sexuality, and religion. Qualitative Psychology, 2(2), 147–163. 10.1037/qup0000026

[bibr39-08862605211072180] Pinel E. C. (1999). Stigma consciousness: The psychological legacy of social stereotypes. Journal of Personality and Social Psychology, 76(1), 114-128. 10.1037//0022-3514.76.1.114 9972557

[bibr40-08862605211072180] Schönfeld P. Brailovskaia J. Margraf J. (2017). Positive and negative mental health across the lifespan: A cross-cultural comparison. International Journal of Clinical and Health Psychology: IJCHP, 17(3), 197–206. 10.1016/j.ijchp.2017.06.003 30487895 PMC6220922

[bibr41-08862605211072180] Spitzer R. L. Kroenke K. Williams J. B. W. Löwe B. (2006). A brief measure for assessing generalized anxiety disorder: The GAD-7. Archives of Internal Medicine, 166(10), 1092–1097. 10.1001/archinte.166.10.1092 16717171

[bibr42-08862605211072180] Stephenson R. Finneran C. (2013). The IPV-GBM Scale: A new scale to measure intimate partner violence among gay and bisexual men. PLoS ONE, 8(6), e62592. 10.1371/journal.pone.0062592 23755098 PMC3674004

[bibr43-08862605211072180] Straus M. Hamby S. Boney-McCoy S. Sugarman D. (1996). The Revised Conflict Tactics Scales (CTS2): Development and preliminary psychometric data. Journal of Family Issues - J FAM ISS, 17, 283–316. 10.1177/019251396017003001

[bibr44-08862605211072180] Straus M. A. Douglas E. M. (2004). A short form of the Revised Conflict Tactics Scales, and typologies for severity and mutuality. Violence and Victims, 19(5), 507–520. 10.1891/vivi.19.5.507.63686 15844722

[bibr45-08862605211072180] Szymanski D. M. (2006). Does internalized heterosexism moderate the link between heterosexist events and lesbians’ psychological distress? Sex Roles, 54(3), 227–234. 10.1007/s11199-006-9340-4

[bibr46-08862605211072180] Szymanski D. M. Chung Y. B. (2001). The Lesbian Internalized Homophobia Scale: A rational/theoretical approach. Journal of Homosexuality, 41(2), 37–52. 10.1300/J082v41n02_03 11482427

[bibr47-08862605211072180] Testa R. J. Habarth J. Peta J. Balsam K. Bockting W. (2014). Development of the gender minority stress and resilience measure. Psychology of Sexual Orientation and Gender Diversity, 2(1), 65. 10.1037/sgd0000081

[bibr48-08862605211072180] Walters M. L. Chen J. Breiding M. J. (2013). The national intimate partner and sexual violence survey: 2010 findings on victimization by sexual orientation. Atlanta, GA: National Center for Injury Prevention and Control, Centers for Disease Control and Prevention. 10.1037/e541272013-001

[bibr49-08862605211072180] World Health Organization (2012). Understanding and addressing violence against women: Intimate partner violence (understanding and addressing violence against women (p. 12). [Information sheet]. World Health Organization. https://www.who.int/reproductivehealth/topics/violence/vaw_series/en/

[bibr50-08862605211072180] Woulfe J. M. Goodman L. A. (2021). Identity abuse as a tactic of violence in LGBTQ communities: Initial validation of the Identity Abuse Measure. Journal of Interpersonal Violence, 36(5–6), 2656–2676. 10.1177/0886260518760018 29528799

